# Osseous Infections Caused by *Aspergillus* Species

**DOI:** 10.3390/diagnostics12010201

**Published:** 2022-01-14

**Authors:** Christos Koutserimpas, Ifigeneia Chamakioti, Konstantinos Raptis, Kalliopi Alpantaki, Georgia Vrioni, George Samonis

**Affiliations:** 1 Department of Orthopaedics and Traumatology, “251” Hellenic Air Force General Hospital of Athens, 11525 Athens, Greece; chrisku91@hotmail.com (C.K.); chamakiotiifigeneia@gmail.com (I.C.); kraptis1981@hotmail.gr (K.R.); 2 Department of Orthopaedics and Traumatology, “Venizeleion” General Hospital of Heraklion, 17121 Heraklion, Greece; apopaki@yahoo.gr; 3 Department of Microbiology, Medical School, National and Kapodistrian University of Athens, 11527 Athens, Greece; gvrioni@med.uoa.gr; 4 Department of Internal Medicine, University Hospital of Heraklion, 71500 Heraklion, Greece

**Keywords:** fungal ostemyelitis, osteoarticular infection, osseous infection, *Aspergillus*

## Abstract

Background: Osteomyelitis caused by *Aspergillus* spp. is a severe, but rare, clinical entity. However, clear guidelines regarding the most effective medical management have not yet been established. The present study is a literature review of all such cases, in an effort to elucidate epidemiology, as well as the therapeutic management and the infection’s outcome. Methods: A thorough review of all reports of osteomyelitis of the appendicular and the axial skeleton, without the skull and the spine, caused by *Aspergillus* spp. was undertaken. Data about demographics, imaging techniques facilitating diagnosis, causative *Aspergillus*, method of mold isolation, antifungal treatment (AFT), surgical treatment, as well as the infection’s outcome were recorded and evaluated. Results: A total of 63 cases of osseous infection due to *Aspergillus* spp. were identified. The studied population’s mean age was 37.9 years. The most commonly affected site was the rib cage (36.8%). Most hosts suffered immunosuppressive conditions (76.2%). Regarding imaging methods indicating diagnosis, computer tomography (CT) was performed in most cases (42.9%), followed by plain X-ray (41.3%) and magnetic resonance imaging (MRI) (34.9%). The most frequent isolated mold was *Aspergillus fumigatus* (49.2%). Cultures and/or histopathology were used for definite diagnosis in all cases, while galactomannan antigen test was additionally used in seven cases (11.1%), polymerase chain reaction (PCR) in four cases (6.3%), and beta-d-glucan testing in three cases (4.8%). Regarding AFT, the preferred antifungal was voriconazole (61.9%). Most patients underwent surgical debridement (63.5%). The outcome was successful in 77.5%. Discussion: Osteomyelitis due to *Aspergillus* spp. represents a severe infection. The available data suggest that prolonged AFT in combination with surgical debridement is the preferred management of this infection, while identification of the responsible mold is of paramount importance.

## 1. Introduction

Fungal osteoarticular infections represent a severe invasive disease caused by hematogenous dissemination, but also by direct inoculation, or direct extension from a nearby infection focus [1,2,3]. Hematogenous spread is the most frequent cause of osseous infection, while direct inoculation is mainly associated with prosthesis implantation or instrumentation. The incidence of such infections is constantly increasing mainly in immunocompromised hosts [2,3]. Nevertheless, they also may more rarely affect immunocompetent patients as well [2]. The main predisposing factors for fungal osteomyelitis are immunosuppression by disease or medications, substance abuse, presence of an indwelling catheter, parenteral hyperalimentation, diabetes mellitus, long-term use of broad-spectrum antimicrobials, HIV infection, and organ transplantation [1,2].

*Aspergillus* spp. are ubiquitous molds causing a wide range of clinical syndromes depending on the immune status of the host [1,2,3]. Immunocompromised patients have increased during the last decades, leading to consecutive increases in fungal invasive infections. Invasive Aspergillosis represents an important cause of morbidity as well as mortality in immunocompromised patients [1]. Aspergillosis of the respiratory system, as well as that of the soft tissues and the skin, due to inhaled spores with infrequent involvement of the gastrointestinal tract, has been well documented [1,3]. Osteomyelitis caused by *Aspergillus* spp. represents a rare and severe opportunistic infection. Most of such cases involve vertebras, presenting as spondylodiscitis [2]. These infections, particularly in children, may occur through contiguous spread, usually from a pulmonary or sinus infection or from the overlying skin. In older patients, the fungus may also spread hematogenously. However, it is of note that growth of *Aspergillus* species in blood cultures is extremely rare [3].

The most pathogenic species among *Aspergilli* is *A. fumigatus*, while twenty other species may cause infection [2,3]. The most common ones are *A. flavus*, *A. terreus*, *A. nidulans*, and *A. niger* [3]. However, an increase in the number of “cryptic” *Aspergillus* species has been identified, such as *A. lentulus N. pseudofischeri*, *A. udagawae*, *A. viridinutans*, *A. fumigatiaffinis*, and *A. novofumigatus* of the *Fumigati* section; *A. alliaceus* of the *Flavi* section; *A. carneus* and *A. alabamensis* of the *Terrei* section; *A. tubingensis*, *A. awamori*, and *A. acidus* of the *Nigri* section; *A. sydowii* of the *Versicolores* section; *A. westerdijkiae* and *A. persii* of the *Circumdati* section; and *A. calidoustus*, *A. insuetus*, and *A. keveii* of the *Usti* section. Nevertheless, the clinical context has been detailed only for a very limited number of these strains and information regarding AFT effectiveness is even more scarce [4].

This type of osteoarticular infection is not well understood [2,3,4,5,6]. Diagnosis and management of osseous invasive aspergillosis represent a real challenge. The rarity and diversity of the disease’s presentation, often lacking an obvious host response to the infection, especially in patients with severe immune deficiencies, make the clinical diagnosis extremely difficult [1,7]. Firm diagnosis, achieved by cultures and/or histopathology, following direct sampling and proper therapy are of paramount importance. All patients require causative antifungal treatment (AFT) and many of them require additional surgical intervention. Surgical debridement is considered the gold-standard of chronic bacterial osteomyelitis management. Debridement of fungal osteomyelitis may also be important and involves the removal of sinus tracts. However, it has been a subject of debate, as some *Aspergillus* osteomyelitis cases that received successful medical treatment did not require surgery [1,2,7]. There are scarce data and limited research has been conducted on surgical management of this infection. Hence, official guidelines on when surgical intervention is necessary do not exist.

*A. fumigatus* is the most common etiologic agent of *Aspergillus* osteomyelitis, being responsible for approximately 80% of these cases. Nevertheless, *A. flavus* and *A. terreus* may also cause such infections [4].

Few *Aspergillus* osteomyelitis cases in the appendicular skeleton may be found in the literature. Therefore, a consensus on diagnostic criteria and the most effective medical management is based on limited data.

The present study is a review of all published cases of *Aspergillus* osteomyelitis in an effort to describe epidemiology, patients’ characteristics, as well as medical and surgical treatment options and their effectiveness. 

## 2. Methods

A thorough electronic search of the PubMed and MEDLINE databases was performed to locate all existing articles related to *Aspergillus* osteomyelitis cases from January 2003 to October 2021. Alone and/or in combination, the terms “*Aspergillus* osteomyelitis”, “fungal osteomyelitis”, “*Aspergillus* osseous infection”, “*Aspergillus fumigatus* osteomyelitis”, “*Aspergillus* bone infection”, and “fungal skeleton infection” were searched. In addition, terms including each *Aspergillus* species (e.g., “*Aspergillus terreus* osteomyelitis”, “*Aspergillus flavus* osteomyelitis”, and so on) were also searched. Following the identification of these reports, individual references from each publication were further reviewed for locating additional cases.

The review was limited to papers published in English and in peer-reviewed journals. Expert opinions; book chapters; studies on animals, on cadavers or in vitro investigations; as well as abstracts of scientific meetings were excluded. This review is also limited to cases published after 2003, as voriconazole, which has been as proven the drug of choice against *Aspergillus* spp. and changed the therapeutic results, was introduced that year. Furthermore, vertebral as well as skull infections were excluded.

The data extracted from these studies included age, gender, location of the osseous infection, responsible *Aspergillus* species, other site of Aspergillosis, co-infection with bacterial species, C-reactive protein (CRP) and erythrocyte sedimentation rate (ESR) at initial presentation, presence of immunosuppressive condition, duration and type of AFT, and type of surgical intervention. Furthermore, the results of medical and surgical treatment, along with the follow-up of each case, were evaluated. Treatment was considered successful if all signs and symptoms of the infection disappeared and no recurrence was observed during the follow-up period.

Data were recorded and analyzed using Microsoft Excel 2019 (Microsoft Corporation, Redmond, WA, USA).

## 3. Results

A total of 63 patients (46 males; 73%), with a mean age of 37.9 years [standard deviation (SD) = 25.3], suffering osteomyelitis due to *Aspergillus* spp. were identified during the study period [1,7,8,9,10,11,12,13,14,15,16,17,18,19,20,21,22,23,24,25,26,27,28,29,30,31,32,33,34,35,36,37,38,39,40,41,42,43,44,45,46,47,48,49,50,51,52,53,54,55,56,57].

A total of 68 osseous infections were recorded because, in five patients, two sites of infection were observed (cases 7, 14, 15, 16, and 42 in Table 1). Regarding the site of infection, the rib cage represented the most commonly affected region (25 cases; 36.8%); followed by the sternum (13; 19.1%); the tibia (7; 10.3%); the femur (5; 7.4%); the ankle and the foot (4 each; 5.9%); the humerus (3 each; 4.4%); the ilium and the scapula (2 each; 2.9%); and the patella, the wrist, and the fibula (1 each; 1.5%).

Mean CRP and ESR upon initial presentation were 49.6 mg/L (SD = 72.9) and 72.4 mm/h (SD = 34.7), respectively. Another site of *Aspergillus* infection was reported in 17 patients (27%). The mean follow-up was found to be 12.2 months (SD = 11.6). Furthermore, 48 patients (76.2%) were immunocompromised according to the available information from each report. The majority of these patients suffered from chronic granulomatous disease (17 cases; 35.4%), followed by patients with diabetes mellitus (12 cases; 25%), organ transplant recipients under immunosuppressive therapy (7 cases; 14.6%), and patients receiving chemotherapy (6 cases; 12.5%). Additionally, it is of note that 10 patients (15.9%) had suffered trauma and/or underwent surgery involving the infected area.

Details on patients’ symptomology are thoroughly presented in Table 1. Pain represented the main complaint in most cases (32; 50.8%), followed by local symptoms of inflammation in 21 (33.3%), pyrexia in 17 (27%), and weight loss in 4 (6.3%).

Regarding imaging methods indicating osseous infection, computer tomography (CT) was performed in 27 patients (42.9%), followed by plain X-ray in 26 (41.3%) and magnetic resonance imaging (MRI) in 22 (34.9%). In 13 cases (cases 5, 21, 23, 27, 29–34, 40, 43, and 48 in Table 1), no imaging was reported.

All osteomyelitis cases due to *Aspergillus* spp. were diagnosed through cultures and/or histopathology. Galactomannan antigen test was additionally used in seven cases (cases 1, 22, 23, 24, 25, 36, and 55 in Table 1), while polymerase chain reaction (PCR) was used in four cases (cases 1, 49, 57, and 59 in Table 1). Furthermore, in three cases (cases 55, 58, and 59 in Table 1), beta-D-glucan testing was additionally performed.

A total of 63 *Aspergillus* spp. strains were isolated. The most commonly isolated was *A. fumigatus* (31 strains; 49.2%), followed by *A. flavus* (13; 20.6%), *A. nidulans* (5; 7.9%), and *A. versicolor* and *A. terreus* (1 each; 1.6%). Additionally, 12 (19%) isolates were not further characterized.

Medical management, as well as the infection’s outcome of the reported cases, are highlighted in Table 2. Regarding AFT, 28 cases (44.4%) were treated with a single antifungal drug, while 18 cases (28.6%) were treated with two, either simultaneously or consecutively, and 15 cases (23.8%) were treated with more than two antifungal agents. Information regarding the specific antifungal drug was not reported in three cases (4.8%) (cases 35, 50, and 54 in Table 2). The mean AFT duration was 5.3 months (SD = 4.9).

Voriconazole was the preferred antifungal, used in 39 cases [(61.9%), in 20 (51.3%) as monotherapy]; followed by amphotericin B in 32 [(50.8%), in 4 (12.5%) as monotherapy]; itraconazole in 21 [(33.3%), in 2 (9.5%) as monotherapy]; caspofungin in 6 [(9.5%), none as monotherapy]; posaconazole in 4 [(6.3%), none as monotherapy]; flucytosine in 3 [(4.8%), none as monotherapy]; fluconazole, micafungin, and isavuconazole in 2 [(3.2%), fluconazole in 1 case (50%) as monotherapy, while the other drugs were given in combination with additional antifungals]; and tioconazole in 1 [(1.6%), as monotherapy].

The infection’s outcome was successful in 48 cases (76.2%), while the mortality rate attributed to the infection and/or its complications was found to be 20.6%. 

Surgical debridement was additionally performed in 40 cases (63.5%). The infection’s outcome in these cases was successful in 31 cases (77.5%), while the mortality rate was 22.5%.

## 4. Discussion

Fungi of the *Aspergillus* species may lead to severe infections in human hosts, including a broad range of clinical presentations, such as aspergilloma (or fungus ball in the lung), severe asthma, sinus fungus ball and granulomatous sinusitis, otomycosis, keratitis, endophthalmitis, skin, wound and osteoarticular infections, chronic pulmonary aspergillosis, tracheobronchitis, invasive pulmonary, and disseminated disease [4,58]. Invasive Aspergillosis represents an important cause of morbidity and mortality in immunosuppressed hosts. It has been estimated that about 200,000 cases of invasive Aspergillosis occur worldwide per annum. However, this does not account for all actual cases, as a lack of accuracy in the diagnosis or treatment has led to increased mortality that ranges between 20% and 100% [4,58].

Osteoarticular Aspergillosis is not a common infection, while, owing to its rarity, it is not well described or understood. Data and information about *Aspergillus* osteomyelitis are scarce [34,39,59]. The present study reviewed all osteomyelitis cases caused by *Aspergillus* spp., reported from 2003 to 2021 in the literature, scoping to elucidate epidemiology, patient’s characteristics, causative *Aspergillus* spp., as well as the medical and surgical treatment and their effectiveness.

Voriconazole is an antifungal agent introduced in 2003 and represents the treatment of choice for invasive Aspergillosis. This agent changed for the better the results of medical AFT. Alternative AFTs consist of lipid or liposomal formulation of amphotericin B and isavuconazole, while other treatments used as salvage therapies are caspofungin, micafungin, anidulafungin, posaconazole, and itraconazole [2,60]. Therefore, the present review is limited to cases published after 2003.

The present study reviewed 63 cases of osteomyelitis, yielding 63 *Aspergillus* spp. strains. The present sample was rather young, as the patients’ mean age was about 38 years, ranging from to 2.5 to 79 years, while the male gender was highly represented (73% males). However, it is of note that another site of *Aspergillus* infection was evident in 17 patients (27%). Therefore, several patients did not suffer from an apparent pulmonary or other extra-osseous *Aspergillus* focus, indicating that isolated *Aspergillus* osteomyelitis may occur de novo. It is also of note that 10 patients (15.9%) had suffered trauma and/or underwent surgery involving the infected area. Hence, it should be kept in mind that non-immunocompromised patients may also be at risk, as previous surgical procedures or trauma may serve as a source of direct inoculation.

In the present review, the majority of affected patients were immunocompromised (76.2%). More particular, most of the hosts suffered from chronic granulomatous disease (35.4%), followed by patients with diabetes mellitus (25%), organ transplant recipients under immunosuppressive therapy (14.6%), and patients receiving chemotherapy (12.5%). Regarding immunosuppressive conditions, there has been documented a spectrum of risk for invasive Aspergillosis. More specifically, conditions having high risk include chronic granulomatous disease, lung or hear transplantation, and leukemia under chemotherapy, while conditions with intermediate risk include liver transplantation, small bowel transplantation, myelodysplastic syndrome, and kidney failure. Finally, clinical entities with low risk include multiple myeloma, chronic obstructive pulmonary disease, non-Hodgkin’s lymphoma, solid tumor, AIDS, and diabetes mellitus [61]. It is believed that approximately 10 million patients with an impaired immune system per year are considered at risk to develop invasive aspergillosis [4].

It should be highlighted that almost 15% of the study’s immunocompromised patients were organ transplant recipients. In these cases, it is important, if such an infection is presumed, to reduce the immunosuppressive treatment, as the degree of immunosuppression strongly influences the outcome of invasive aspergillosis. Furthermore, it should be taken into consideration that voriconazole, the antifungal drug of choice, interferes with the P450 cytochrome oxidase [1,14,35]. Hence, this agent may alter the activity and the levels of some immunosuppressive drugs [14,35]. 

The onset of fungal infections is often insidious with non-specific symptoms, thus diagnosis is often a challenge [35,62,63,64]. As highlighted in Table 1, pain, local signs of infection, and fever represent the main symptoms of osteomyelitis caused by *Aspergillus* spp. Such symptoms are common clinical indicators for most osseous infections. Additionally, no other specific clinical manifestation exists that could consistently differentiate between bacterial and *Aspergillus* osteomyelitis. Therefore, the laboratory firm diagnosis is of utmost important for the identification of the causative microorganism. Early recognition of *Aspergillus* osteomyelitis plays a major role in the infection’s outcome.

Systemic inflammatory markers, such as CRP and ESR, which represent markers mainly used in every-day clinical practice, may be minimally elevated or even normal in cases of fungal osteomyelitis [4,61,62]. Hence, a detailed medical history, focused on potential immunosuppressive conditions and/or medications, as well as a thorough physical examination, are very important. In the present study, mean CRP and ESR upon initial presentation were found to be 49.6 mg/L and 72.4 mm/h, respectively.

Imaging techniques also play an important role facilitating the diagnosis. In the present study, CT was performed in most cases (42.9%), followed by plain X-ray (41.3%) and MRI (34.9%). In plain X-rays, suspicious signs for osteomyelitis include cortical erosion, permeative marrow lucency and periosteal reaction or sclerosis, and cortical thickening. CT scans demonstrate subtle cortical erosions earlier than radiographs and reveal sequestra [65]. MRIs reveal typical findings of osteomyelitis, including edema and enhancement of bone marrow, along with the replacement of bright fatty marrow signal on T1 weighted images with signal closer to the muscle intensity. T2 marrow hyperintensity and enhancement, as well as T2 hyperintense periosteal edema, may be reactive to adjacent soft tissue infection. Hence, T1 marrow replacement is the most specific sign of marrow infection [65].

The present review has revealed that the most common site of osteomyelitis due to *Aspergillus* spp. is the rib cage (36.8%), followed by the sternum (19.1%), the tibia (10.3%), and the femur (7.4%). Other sites, such as the ankle, the foot, the humerus, the ilium, the scapula, the patella, the wrist, and the fibula, were also identified, but not highly represented.

*Aspergilli* are relative common food and soil contaminants, while their spores are ubiquitous [4,58,62]. The most frequent species involved in human infection is *Aspergillus fumigatus* [2,58,62]. Although *A. fumigatus* is the most common etiologic agent, being responsible for approximately 80% of the cases of Aspergillosis, *A. flavus* and *A. terreus* may also cause such infections. In the present study, *A. fumigatus* was the most common isolated one (49.2%), while *A. flavus* (20.3%), *A. nidulans* (7.9%), and *A. versicolor* and *A. terreus* (1.6% each) followed.

In all cases of the present study, the causative *Aspergillus* strain was isolated from clinical specimens and was identified with cultures and/or histopathology, while galactomannan antigen test was additionally used in seven patients (11.1%) and PCR in four patients (6.3%). Additionally, in three patients (4.8%), beta-d-glucan testing was additionally performed.

Conventional identification methods of *Aspergillus* spp. include macroscopic as well as microscopic morphological characteristics [61,62]. Furthermore, additional methods include serum beta-d-glucan, which is a component of the fungal cell wall, PCR testing, and galactomannan antigen test. Serum beta-d-glucan has proved to be helpful. However, this marker does not possess specificity for *Aspergillus* spp., thus its presence may indicate other invasive fungal infections as well. Galactomannan antigen test provides, mainly, prognostic information with declining values related to effective AFT. PCR testing has not been widely adopted owing to a lack of standardization [4,61].

In all the present cases, the mold was isolated with conventional techniques, while additional methods were also performed in a few cases. Regarding cultures, *Aspergillus* spp. grow generally well on standard media. However, the use of fungal-specific media may increase this mold’s isolation. In some cases, it represents a real challenge to determine the clinical significance of positive cultures from nonsterile sites, while medical history, risk factors, and imaging findings may be extremely helpful. It must be taken into account, though, that AFT may decrease the cultures’ yield. Regarding histopathology, direct visualization of the branching septate hyphae on microscopic examination along with recovery of the organism in cultures provide a definite diagnosis [2,4,61].

A major issue in this infection’s management is the development of resistance of *Aspergillus* to antifungal agents, including the azole compounds [6,62,66,67]. Owing to over-use of pesticides containing azoles, mainly in the Netherlands and England, an increased azole resistance has been observed in these countries. Nevertheless, in other countries, this has not yet become an issue [61]. Hence, it is of paramount importance to identify the mold and its susceptibilities and to attain accurate MIC values. The EUCAST has standardized antifungal clinical breakpoints for several methods measuring MICs. However, it should be noted that the immune condition of the host is the most critical issue regarding the success of antifungal medical management [68,69].

The present study has shown that, in the majority of cases, voriconazole was the preferred drug, used in 39 cases (61.9%), either as monotherapy or in combination with another antifungal, while amphotericin B was used in 32 cases (50.8%).

Triazoles and amphotericin B represent the antifungal drugs proposed for the management of invasive Aspergillosis, possessing cidal action, while echinocandins have also been used, possessing only static effects. The triazoles exert their antifungal effects by inhibiting the cytochrome P450 (CYP)-dependent 14-a-demethylase, blocking the conversion of lanosterol to ergosterol. Voriconazole, an agent that possesses the features of azole compounds, such as moderate hepatotoxicity and minor nephrotoxicity, as compared with all amphotericin formulations, was introduced and approved in 2003 as the antifungal regimen of choice against invasive aspergillosis [60,67]. Voriconazole is a triazole with a broad spectrum of antifungal activity. Its efficacy against all *Aspergilli* of clinical importance is this drug’s most important characteristic. In particular, this antifungal targets the synthesis of ergosterol biosynthesis by inhibition of the cytochrome P450-dependent enzyme lanosterol 14-alpha-demethylase, resulting eventually in severe cell membrane damages and, consequently, inhibition of the fungal cell growth and replication or death. This drug also inhibits enzymes associated with the function of the P450 cytochrome, regulating the functional respiration chain [1,62]. This drug’s serum concentrations are significantly influenced by a broad range of other drugs owing to extensive drug to drug interactions. Voriconazole is available in oral and intravenous preparations [60,62]. Its adverse effects include liver function test abnormalities (15% of patients), gastrointestinal toxicities (nausea, vomiting, diarrhea), as well as skin rashes. There have also been other, less common, complications described, such as hallucinations, visual abnormalities (mostly related to higher serum concentrations, transient, and reversible), and fluorosis or cutaneous malignancy with long-term administration. It is important to closely monitor dosing in adults, as the drug’s levels are unpredictable, owing to a variety of factors, including gender, age, liver disease, and potential genetic polymorphisms in CYP2C19 [60,62,67].

Amphotericin B is a very broad-spectrum antifungal agent that has been used for decades. It is a polyene that is fungicidal and seems to act by creating extra-membranous masses that extract ergosterol from lipid bilayers, leading in cell death and resulting in the development of ion channels, associated with destruction of the fungal cells. Its main problem, prohibiting its long-term use, is the drug’s nephrotoxicity and the associated electrolyte disturbances [2,70]. The liposomal formulations of amphotericin B are less nephrotoxic [70]. In the present study, exact information about the amphotericin B type was not available in most of the reports. Nevertheless, it is reasonable to assume that amphotericin B lipid or liposomal formulations have been the drugs of choice during the last two decades, because, during this period, the therapeutic use of deoxycholate amphotericin B has been abandoned.

Itraconazole is an azole available for per os and iv administration. Given per os may be problematic, as its absorption is erratic, making its use difficult for severe infections. Adverse effects include nausea, vomiting, hypertriglyceridemia, hypokalemia, and hepatotoxicity [61]. Furthermore, negative inotropic effects have also been reported, thus it should be used with extreme caution in patients suffering heart failure. Itraconazole may be mainly used in noninvasive or chronic forms of aspergillosis, or following intolerance or toxicity to other triazoles [61,62]. In the present study, itraconazole was the preferred antifungal drug in 21 cases (33.3%), while in most cases (90.5%), it was used in combination with another antifungal drug.

Posaconazole is another triazole with an analogous structure to itraconazole available in per os and iv formulations. It is highly active in vitro against *Aspergillus* spp. Posaconazole is metabolized in the liver through glucuronidation and has drug to drug interactions involving of the azole compounds. It is mainly used as prophylaxis in severe immunocompromised, high-risk patients undergoing bone marrow transplant with graft versus host disease and in patients with acute myelogenous leukemia and myelodysplastic syndrome [61]. In the present study, posaconazole was used in four patients (6.3%) in combination with another antifungal agent.

Isavuconazole is effective against *Aspergillus* spp. It has been compared to voriconazole and was revealed to be noninferior and to have 17% fewer complications. It is available in oral and intravenous forms, while adverse effects include nausea, vomiting, and diarrhea [51,60]. Isavuconazole was used, in the present study, in just two cases (3.2%), in combination with another antifungal regimen.

The echinocandins, including caspofungin, anidulafungin, and micafungin, inhibit the synthesis of 1,3-b-d-glucan via the glucan synthase enzyme. All are available in iv formulations. They are generally well tolerated, but they are fungistatic, rather than fungicidal [61]. In the present study, echinocandins were used in eight cases (12.7%), in combination with other antifungal drugs.

Prolonged AFT is crucial for the treatment of these infections. The mean AFT duration in the present study was 5.3 months.

Invasive Aspergillosis, including osteomyelitis, is a severe infection with high mortality, especially in immunosuppressed hosts, despite the use of effective antifungal drugs [61]. The severity of this fungal infection is portrayed through the relative high mortality rate (20.6%). Thus, in many cases, combination therapy is given, although such guidelines do not exist. This also becomes apparent by the findings of the present review, because, in 33 cases (52.4%), two or more antifungal agents were used for the eradication of the infection.

Fungal osteomyelitis also demands, in most cases, surgical debridement. In the present review, most patients (63.5%) underwent debridement. Surgical debridement includes thorough removal of the sequestrum and the sinus tracts. 

The present study has some limitations. Not all information from a number of the reviewed cases was available. Hence, dosages, drug serum-levels, MICs, and side effects of the used antifungal drugs, in most cases, were not described. However, this review provides valuable information about epidemiology, symptomatology, diagnosis, medical and surgical management, as well as outcome of cases of osteomyelitis caused by *Aspergillus* spp.

In conclusion, osteomyelitis caused by *Aspergillus* spp. represents a severe and, in many cases, life-threatening infection, as it affects mainly, but not exclusively, immunocompromised hosts. This infection demands prompt diagnosis and early multidisciplinary management, because, in addition to medical treatment, most cases require surgical intervention. Although new techniques, such as PCR testing, have been developed, conventional methods including cultures and histopathology remain the main tools of isolating the causative mold. Prolonged AFT, guided by susceptibility tests, along with surgical debridement represent the most effective therapeutic approach. Additionally, in culture negative for bacteria and/or cocci osteomyelitis cases, a high index of suspicion for fungal pathogens should be present, especially in immunocompromised hosts.

## Figures and Tables

**Table 1:** Main characteristics of the published osteomyelitis cases due to *Aspergillus* spp. Year of publication, patient’s demographics, responsible *Aspergillus* spp., site of infection, immunosuppressive condition and/or medications, other site of Aspergillosis, and symptoms. M: male, F: female, CGD: chronic granulomatous disease, TBC: tuberculosis, LT: lung transplant, RT: renal transplant, IST: immunosuppressive treatment, DM: diabetes mellitus, HT: heart transplant, LSI: local signs of inflammation.

Case No	Year	Reference	Gender/Age	*Aspergillus* Species	Location	Previous Surgery or Trauma of the Affected Region	Immunosuppressive Conditions and/or Medications	Symptoms
1.	2003	[8]	M/16	*A nidulans*	femur	-	CGD	Pain, pyrexia
2.	2003	[9]	M/12	spp.	ilium	Yes	CGD	Pain, restriction of ROM, pyrexia
3.	2003	[10]	M/17	*A fumigatus*	patella	-	TBC, antituberculosis therapy	Pyrexia, lymphadenopathy
4.	2003	[11]	F/13	spp.	ilium	-	Leukemia, chemotherapy	Pyrexia, pain
5.	2003	[12]	F/8	*A fumigatus*	rib cage	-	CGD	-
6.	2004	[13]	M/48	*A fumigatus*	rib cage	-	Heroin abuse, methadone replacement	Pain, weight loss
7.	2004	[14]	M/64	*A fumigatus*	foot, ankle	-	Bilateral LT recipient, IST	LSI, pain, pyrexia
8.	2004	[15]	M/74	*A flavus*	sternum	-	Renal failure	Fatigue, malaise, pyrexia LSI, sterno-cutaneous fistula
9.	2004	[15]	M/63	*A flavus*	sternum	-	Chronic obstructive pulmonary disease	Fatigue, malaise, pyrexia LSI, sterno-cutaneous fistula
10.	2004	[15]	M/56	*A flavus*	sternum	-	DM, asthma	Fatigue, malaise, pyrexia LSI, sterno-cutaneous fistula
11.	2005	[16]	M/65	spp.	rib cage	-	Heavy smoker, alcoholism, TBC	Pain, weight loss
12.	2005	[17]	M/40	*A versicolor*	foot	-	Acute lymphoblastic leukemia, chemotherapy, renal failure	Pain, LSI, pyrexia
13.	2005	[18]	M/62	*A fumigatus*	rib cage	Yes	DM, chronic obstructive pulmonary disease	Pain, LSI
14.	2005	[19]	M/5	*A fumigatus*	rib cage and wrist	-	-	LSI, pain
15.	2005	[20]	M/43	*A fumigatus*	femur and fibula	Yes	-	-
16.	2005	[20]	M/31	*A fumigatus*	rib cage	-	AIDS	-
17.	2005	[20]	M/4	*A fumigatus*	rib cage	-	CGD	-
18.	2005	[20]	F/4	*A nidulans*	sternum, femur	-	CGD	-
19.	2005	[20]	M/14	*A fumigatus*	rib cage	-	CGD	-
20.	2005	[20]	M/9	*A fumigatus*	rib cage	-	Acute lymphocytic leukemia	-
21.	2007	[21]	M/8	*A fumigatus*	rib cage	-	CGD	-
22.	2007	[22]	M/39	*A fumigatus*	sternum	-	TBC, AIDS, hepatitis B	Pain
23.	2007	[23]	F/29	*A terreus*	sternum	-	DM, RT recipient, IST	LSI
24.	2007	[24]	M/34	*A fumigatus*	ankle (talar bone)	-	RT recipient, hepatitis C, IST, DM, TBC	LSI, pain, restriction of ROM
25.	2008	[25]	M/21	*A nidulans*	rib cage	-	CGD	LSI
26.	2008	[26]	M/60	*A fumigatus*	sternum	-	DM, smoker, heart failure	LSI, sterno-cutaneous fistula
27.	2008	[27]	-/14	spp.	rib cage	-	CGD	-
28.	2008	[28]	M/69	*A flavus*	tibia	Yes	DM	LSI, ulcer
29.	2009	[29]	M/70	*A flavus*	rib cage	-	-	Pain
30.	2009	[30]	M/18	*A fumigatus*	foot	-	CGD	-
31.	2009	[31]	F/60	*A fumigatus*	humerus	-	RT and pancreas transplant recipient, IST	-
32.	2009	[31]	F/62	*A fumigatus*	rib cage	-	Lung cancer (remission), chemotherapy	-
33.	2009	[31]	M/63	*A fumigatus*	sternum	-	-	-
34.	2009	[31]	F/79	*A fumigatus*	sternum	-	-	-
35.	2010	[32]	M/4	spp.	femur	-	Acute lymphatic leukemia, chemotherapy	Pyrexia, pain
36.	2011	[33]	F/79	*A fumigatus*	femur	-	Lymphocytic leukemia, chemotherapy	Pain
37.	2011	[34]	F/13	*A flavus*	tibia	Yes	-	Pain, restriction of ROM
38.	2011	[35]	M/37	spp.	humerus	-	RT recipient, IST, hepatitis C	Pain, weight loss
39.	2012	[36]	F/2.5	spp.	tibia	-	CGD	LSI, pyrexia
40.	2012	[37]	M/13	*A nidulans*	tibia		CGD	-
41.	2012	[38]	F/72	*A fumigatus*	humerus	Yes	DM, heart failure	Pain
42.	2013	[39]	M/69	*A fumigatus*	sternum and rib cage	-	-	LSI
43.	2013	[40]	M/54	*A flavus*	rib cage	-	DM, renal failure, heart failure	Pain, pyrexia
44.	2013	[41]	F/45	*A fumigatus*	rib cage	-	DM	Pain, weight loss
45.	2013	[42]	F/30	spp.	foot	Yes	-	LSI
46.	2014	[43]	M/28	*A flavus*	tibia	-	-	LSI, pain
47.	2015	[44]	F/26		tibia	Yes	-	LSI, pain, drainage, restriction of ROM
48.	2015	[45]	M/18	*A flavus*	rib cage	-	Acute lymphoblastic leukemia B	-
49.	2015	[46]	M/68	*A flavus*	ankle (calcaneus)	-	DM	Pyrexia, LSI, ulcer
50.	2016	[47]	M/4	spp.	tibia	-	CGD	Pain
51.	2016	[48]	F/4.5	*A fumigatus*	rib cage	-	CGD, TBC	Pyrexia, pain
52.	2017	[49]	M/51	*A flavus*	rib cage	-	-	Pain, pyrexia
53.	2017	[49]	M/40	*A flavus*	rib cage	Yes	-	Pain, pyrexia
54.	2018	[50]	M/7	*fumigatus*	ankle (talar bone)	-	CGD	Pain, drainage
55.	2018	[51]	M/70	*fumigatus*	sternum	-	Chronic obstructive pulmonary disease	Pain
56.	2018	[7]	M/9	spp.	scapula	-	CGD	Pain, pyrexia
57.	2019	[52]	F/61	*A flavus*	rib cage	-	Smoker, breast cancer, radiotherapy, chronic obstructive pulmonary disease, lymphoma, chemotherapy	Pain
58.	2020	[53]	M/65	*A fumigatus*	sternum	Yes	HT recipient, IST	LSI
59.	2020	[54]	M/37	*A fumigatus*	rib cage	-	-	LSI
60.	2020	[55]	M/10	*A nidulans*	rib cage	-	CGD	Pain, night sweets, lethargy, coryza, weakness
61.	2020	[1]	M/52	*A fumigatus*	scapula	-	HT recipient, IST	Pain
62.	2020	[56]	F/65	spp.	sternum	-	DM, heart failure	LSI
63.	2021	[57]	M/77	*A fumigatus*	rib cage	-	Rheumatoid arthritis, chronic obstructive pulmonary disease, DM	Pain, drainage

**Table 2:** Therapeutic management of osteomyelitis due to *Aspergillus* spp. Antifungal treatment (AFT), duration of AFT, and infection’s outcome are presented. (*): death as a result of infection.

Case #	Reference	AFT	Total Duration of AFT (Months)	Infection’s Outcome
1.	[8]	Amphotericin B, itraconazole	undefined long-duration	Success
2.	[9]	Amphotericin B	1.5	Success
3.	[10]	Amphotericin B, itraconazole	6	Success
4.	[11]	Voriconazole	6	Success
5.	[12]	Amphotericin B, itraconazole	3.5	Failure *
6.	[13]	Itraconazole	18	Success
7.	[14]	Itraconazole, amphotericin B, posaconazole	12	Success
8.	[15]	Amphotericin B, itraconazole	1	Success
9.	[15]	Amphotericin B, itraconazole	3	Failure *
10.	[15]	Amphotericin B, itraconazole	3	Success
11.	[16]	Amphotericin B	-	Failure *
12.	[17]	Amphotericin B, fluconazole, itraconazole	2	Failure *
13.	[18]	Itraconazole	6	Success
14.	[19]	Amphotericin B, itraconazole	undefined long-duration	Success
15.	[20]	Amphotericin B, itraconazole, voriconazole	3	Success
16.	[20]	Amphotericin B, 5-flucytosine, itraconazole, voriconazole	3	Success
17.	[20]	Amphotericin B, voriconazole	6	Success
18.	[20]	Amphotericin B, itraconazole, 5-flucytosine, voriconazole	6	Success
19.	[20]	Amphotericin B, itraconazole, voriconazole	1	Failure *
20.	[20]	Amphotericin B, voriconazole	2.5	Success
21.	[21]	Amphotericin B, itraconazole	1	Success
22.	[22]	Voriconazole	-	Success
23.	[23]	Amphotericin B, itraconazole, caspofungin, voriconazole	2	Success
24.	[24]	Amphotericin B	1.5	Success
25.	[25]	Amphotericin B, caspofungin, voriconazole	5	Failure *
26.	[26]	Voriconazole	0.5	Success
27.	[27]	Amphotericin B, itraconazole	-	Failure *
28.	[28]	Voriconazole	7	Success
29.	[29]	Voriconazole	6	Success
30.	[30]	Voriconazole, caspofungin, posaconazole, itraconazole	12	Success
31.	[31]	Voriconazole, posaconazole, micafungin	2.5	Failure *
32.	[31]	Voriconazole	0.5	Success
33.	[31]	Fluconazole	0.5	Failure *
34.	[31]	Voriconazole	1	Success
35.	[32]	Two antifungal agents	6	Success
36.	[33]	Amphotericin B, voriconazole	-	Success
37.	[34]	Voriconazole, caspofungin	12	Success
38.	[35]	Voriconazole	2	Success
39.	[36]	Voriconazole	3	Success
40.	[37]	Voriconazole	1	Success
41.	[38]	Voriconazole	18	Success
42.	[39]	Voriconazole	6	Success
43.	[40]	Amphotericin B, itraconazole	6	Success
44.	[41]	Voriconazole	-	Success
45.	[42]	Amphotericin B	1.5	Success
46.	[43]	Amphotericin B, voriconazole	3	Success
47.	[44]	Tioconazole	0.75	Success
48.	[45]	Caspofungin, voriconazole		Success
49.	[46]	Amphotericin B, voriconazole	4	Success
50.	[47]	-	-	-
51.	[48]	Amphotericin B, itraconazole, 5-fluorocytosine	2	Failure *
52.	[49]	Voriconazole	4	Success
53.	[49]	Voriconazole	3	Success
54.	[50]	-	-	Failure *
55.	[51]	Voriconazole	6	Success
56.	[7]	Voriconazole	3.5	Success
57.	[52]	Voriconazole	8.6	Success
58.	[53]	Amphotericin B, voriconazole, isavuconazole	10	Success
59.	[54]	Amphotericin B, voriconazole, posaconazole	9	Success
60.	[55]	Amphotericin B, voriconazole, caspofungin	12	Failure *
61.	[1]	Voriconazole	24	Success
62.	[56]	Amphotericin B, micafungin, voriconazole, isavuconazole,	6	-
63.	[57]	Voriconazole	4	Failure *
